# Urine *N*-Acetylaspartate Distinguishes Phenotypes in Canavan Disease

**DOI:** 10.1089/hum.2024.168

**Published:** 2025-01-16

**Authors:** Amanda Nagy, Florian Eichler, Annette Bley, Janna Bredow, Alexander Fay, Elise L. Townsend, Beth Leiro, Adam Shaywitz, Genevieve Laforet, Danielle Crippen-Harmon, Rachel Williams

**Affiliations:** ^1^ Department of Neurology, Massachusetts General Hospital, Boston, Massachusetts, USA; ^2^ University Medical Center Hamburg-Eppendorf, Leukodystrophy Clinic at University Children’s Hospital, Hamburg, Germany; ^3^ Department of Neurology, University of California San Francisco, San Francisco, California, USA; ^4^ School of Health and Rehabilitation Sciences, MGH Institute of Health Professions, Boston, Massachusetts, USA; ^5^ BridgeBio Gene Therapy, Palo Alto, California, USA.

**Keywords:** Canavan disease, aspartoacylase (ASPA), *N*-acetylaspartic acid (NAA), biomarker, phenotype, genotype

## Abstract

Canavan disease (CD) is an ultra-rare autosomal recessive leukodystrophy caused by loss-of-function mutations in *ASPA,* which encodes aspartoacylase (ASPA), leading to accumulation of *N*-acetylaspartate (NAA). Patients with CD typically present with profound psychomotor deficits within the first 6 months of life and meet few motor milestones. Within CD a subset of patients exhibits a milder phenotype with more milestone acquisition, possibly related to greater residual ASPA activity. An ongoing CD natural history study and a literature search were leveraged to compare urine NAA levels and associated genotypes in patients classified with mild or typical CD, with the hypothesis that urine NAA levels reflect ASPA activity and therefore can distinguish between the two phenotypes. Urine NAA levels were lower, on average (*p* < 0.0001), in individuals with mild (mean 525.3, range 25.2–1,335 mmol/mol creatinine [Cr]) compared with typical CD (mean 1,369, range 391.7–2,420 mmol/mol Cr). Mutations R71H and Y288C, variants that may harbor residual ASPA activity, were unique to the mild phenotype population (56%, 14/25) and not found in individuals with a typical phenotype (0%, 0/39). In aggregate, urine NAA levels can distinguish between mild and typical CD phenotypes, suggesting the ability to reflect ASPA activity.

## INTRODUCTION TO CANAVAN DISEASE

Canavan disease (CD) is a rare monogenic neurodevelopmental disorder caused by autosomal recessive loss-of-function mutations in the *ASPA* gene encoding aspartoacylase (ASPA).^1^ ASPA is expressed by oligodendrocytes in the central nervous system (CNS) where it hydrolyzes *N*-acetylaspartate (NAA), its only known substrate, into acetate and aspartate.^1,2^ No other enzyme is known to hydrolyze NAA and the high levels of NAA observed in CD are considered pathognomonic.^1^

CD is characterized by profound developmental impairment starting in infancy, ultimately leading to death in the first 1–2 decades of life.^3,4^ Children with CD experience developmental arrest at the level of a 3–5-month-old infant.^3,4^ In the rare instance that basic motor skills are achieved (*e.g.*, head control, hand/arm use, supported sitting), they are typically lost in the first years of life.^3,4^

Although CD usually has a severe phenotype, a milder form has been described. This atypical form of CD also has its onset in the first years of life, but is less extreme, with individuals achieving more advanced motor milestones such as walking (independently or with support); regression is not seen until later in childhood or adolescence.^3,5–14^

Associations between genotype and phenotype (biochemical and clinical) in CD remain incompletely characterized; published studies have been limited by small sample size and variable biochemical methodologies.^5,7–22^ Here, we utilize preliminary findings from the CAN*inform* CD natural history study and a literature search to demonstrate that on a population level urine NAA can distinguish between phenotypes, demonstrating a relationship between urine NAA and ASPA activity.

## METHODS

### Natural history study

An ongoing natural history study (NCT04126005, CAN*inform*) is being conducted alongside a Phase 1/2 gene therapy trial for CD (NCT04998396, CAN*aspire*). Due to the ongoing nature of this study, the data presented in this article are preliminary and conclusions may change upon completion of the study.

### Motor milestone assessment

For these analyses, retrospective and/or prospective motor milestone data from 59 natural history study participants were used as a proxy for clinical phenotype. Retrospective Centers for Disease Control and Prevention (CDC) milestone achievement was assessed by a chart review using an edited list of milestones representing key CD concepts in the 2–18-month age range (Supplementary Table S1).^23^ Available data were limited to what was reported in the medical record. Prospective CDC milestone achievements were assessed as “absent” or “present” either remotely or in person up to age 5 years using a combination of trained motor rater observation and caregiver reports. Of note, not all participants had data for some milestones given the variability in what was reported in medical records and the standardized protocol for age-appropriate prospective collection of CDC milestones.

### Urine NAA in natural history study

Urine NAA results were collected for 34/59 participants; in addition, predose results from the interventional trial (CVN-102, NCT04998396) were included, for a total of 38 individuals contributing at least one urine NAA result. Of the 38 individuals with urine NAA results, 27 were prospective and 11 were retrospective from the medical records (Table 1). Natural history participants without NAA results were not included in Table 1 or in the analysis of the relationship between NAA, phenotype, and genotype.

**Table 1.** Urine *N*-acetylaspartate and Genotype Results

Urine NAA and Genotype Results							
Source	Sample ID	Age (Years)	Urine NAA (mmol/mol Cr)	Phenotype	Genotype	Achieved Skill “Walking/Walking with Aid”	Urine NAA method
Cakar et al.^8^	Cakar	13	454.7	Mild	G274R	14 months	Method unknown
Delaney et al.^9^	Delaney	0.33	226.7	Mild	A305E + L30V	At 4 years is running and kicking a ball	Method unknown
		0.58	604.6	Mild			
Sarret et al.^5^	Sarret	1.51	1,308	Mild	A305E + P183H	9 years	Method unknown
Yalcinkaya et al.^17^	Yalcinkaya	2.25	1,335	Mild	A305E + Y288C	6 years noted to walk and jump	Method unknown
Nguyen et al.^21^	Nguyen	7	25.2	Mild	R71H + Y231X	Not specifically mentioned but can be reasonably assumed after notation at 7 years and 10 years that neurological exam was essentially normal, not much difficulty with coordination	Method unknown
Velinov et al.^13^	Velinov	2.08	370	Mild	R71H	19 months	Method unknown
Toft et al.^12^	Toft_Male Patient	2	660	Mild	A305E^a^	18 months	GC
	Toft_Female Patient	6	113	Mild	A305E^a^	20 months	
Surendran et al.^22^ and Benson et al.^19^	Surendran	6	357	Mild	R71H + Y288C	Reasonably assumed as only noted mild developmental delay at age 13 and no sign of neurological regression at age 31	Stable isotope dilution
		13	64.5				
		23	95.6				
		23	101.3				
Janson et al.^10,24^	Janson_Patient 1	4	303	Mild	A305E + R71H	18 months	Method unknown
	Janson_Patient 2	1.58	615	Mild	A305E + R71H	17–19 months	
Tacke et al.^11^	Tacke	4	186.9	Mild	G274R + K213E	4 years	GC
Zafeiriou et al.,^14^ and Tacke et al.^11^	Zafeiriou_Patient 1	7		Mild	Y288C + F295S	16 months	GC
	Zafeiriou_Patient 2	5		Mild	Y288C + F295S	12 years, walks with support	
Kotambail et al.^7^	Proband 1	2.25	400.5	Mild	G176S	3 years, walks with support	Method unknown
	Proband 2			Mild		2 years, ataxia of gait	
Jauhari et al.^20^	Jauhari	5		Mild	Y288C	5 years	Method unknown
Zhou et al.^18^	Zhou	9	480.6	Mild	R168H + E40K	2 years	Method unknown
Gowda et al.^25^	Gowda	0.83	391.7	Typical	A287T		GC
Merrill et al.^26^	Merill	0.19		Typical	A305E		Method unknown
Madhavarao et al.^27^	Madhavarao_Female Patient	1.13	1,950	Typical	E285A		GC
		1.25	1,530				
		1.4	2,580				
	Madhavarao_Male Patient	0.73	2,400	Typical	IVS4 + 1G>T		
		1	2,440				
Solsona et al.^28^	Solsona	0.25	2,579	Typical	C218X		GC
		1.92	663				
Di Pietro et al.^29^	Di Pietro_Older Sibling	4	680.1	Typical	I177T		High Performance Liquid Chromatography
	Di Pietro_Younger Sibling	0.33	1,378.5	Typical	I177T		
Hamaguchi et al.^30^	Hamaguchi	4	585.2	Typical	I143T		GC
CVN-101	1102	0.12	448.2	Typical	A305E		Retrospective, method unknown
CVN-101	1103	0.06	1,076	Typical	A305E		Retrospective, KKI
CVN-101	1106	3.63	1,244	Typical	c.17p13.2 (3357215_3386275)		Retrospective, method unknown
CVN-101	1107	0.79	1,376	Typical	A305E + c.634 + 886_634 + 887insSVA		Retrospective, method unknown
		4.04	1,301.8				Prospective, KKI
		5.16	1,297.9				Prospective, KKI
CVN-101	1108	0.36	1,149.7	Mild	A305E + E285A	Walks holding on	Retrospective, KKI
		5.86	767.1				Prospective, KKI
CVN-101	1113 (102-101-001)	2.23	2,439.5	Typical	N54K		Prospective, KKI
		2.42	1,517.4				Prospective, KKI
CVN-101	1126	0.70	1,195	Typical	E285A + P181T		Retrospective, method unknown
CVN-101	1139	0.92	1,106.8	Typical	A305E + R168H		Prospective, KKI
		1.41	1,398.6				Prospective, KKI
CVN-101	1145 (102-101-003)	1.63	1,087.4	Typical	E285A + I16T		Prospective, KKI
		1.71	1,123.5				Prospective, KKI
		1.82	1,060.3				Prospective, KKI
CVN-101	1154 (102-104-006)	0.58	1,356	Typical	E285A + R168C		Prospective, KKI
		0.69	1,198.4				Prospective, KKI
		0.77	1,173.9				Prospective, KKI
CVN-101	1205	3.39	1,485.1	Typical	no information		Prospective, KKI
CVN-101	1209	0.67	1,569.5	Typical	A305E		Retrospective, method unknown
CVN-101	1210	3.56	1,135.2	Typical	E285A		Prospective, KKI
		4.15	1,202.3				Prospective, KKI
		4.53	1,566.5				Prospective, KKI
		5.07	1,202.3				Prospective, KKI
CVN-101	1211	3.56	1,316	Typical	E285A		Prospective, KKI
		4.15	1,208.8				Prospective, KKI
CVN-101	1232 (102-101-002)	1.21	1,845.5	Typical	A305E + S108L FS*2		Prospective, KKI
		1.52	1,969.4				Prospective, KKI
CVN-101	1234	2.54	747.9	Mild	G274R + A202P	Walked alone at 5.3 years old	Retrospective, method unknown
		8.07	435.2				Prospective, KKI
CVN-101	1235	0.50	662.2	Mild	G274R + A202P	Walked alone at 2.7 years old	Retrospective, method unknown
		6.03	572.1				Prospective, KKI
CVN-101	1317	4.45	1,374.1	Typical	A305E		Prospective, KKI
CVN-101	1318	0.58	1,201	Typical	E285A + unknown		Retrospective, method unknown
CVN-101	1319	0.18	1,473	Typical	A305E + c. 785T > C		Retrospective, method unknown
CVN-101	1322	12.01	785.2	Typical	Y231X + (P181T and Y142X)		Prospective, KKI
CVN-101	1323	18.92	426.2	Typical	E285A + A305E		Prospective, KKI
CVN-101	1328	2.69	1,992.7	Typical	A305E + c.744 + 1G>A		Prospective, KKI
		3.71	1,707.3	Typical			Prospective, KKI
CVN-101	1329	9.58	919.5	Typical	c.878_880delAAG + unknown		Prospective, KKI
CVN-101	1330	9.98	1,040.9	Typical	c.292del		Prospective, KKI
CVN-101	1333	1.33	1,888.7	Typical	A305E		Retrospective, method unknown
CVN-101	1337	3.89	1,375.4	Typical	L247P + unknown		Prospective, KKI
		5.05	1,680.1				Prospective, KKI
CVN-101	1338	4.19	579.9	Mild	Y288C + unknown	Enrolled at 4.2 years old (also noted to run and kick a ball at this age)	Prospective, KKI
CVN-101	1341	0.60	1,338	Typical	c.634 + 1G>T + c.815T>C		Retrospective, method unknown
CVN-101	1351	12.18	258.3	Mild	R71H + P181T	Enrolled at 12 years old with walking, running, somersault, and climbing capabilities	Prospective, KKI
		13.28	240.7				Prospective, KKI
CVN-101	1352 (102-105-007)	0.64	2,177.3	Typical	A305E		Prospective, KKI
		1.32	1802.8				Prospective, KKI
		1.4	1,835.1				Prospective, KKI
CVN-101	1353	1.33	1,988.8	Typical	E293*FS + M195K		Prospective, KKI
CVN-101	1357	0.38	687	Mild	A305E + R71H	Walking at 1.25 years old	Prospective, KKI
CVN-102	1358 (102-101-009)	0.83	1,943.6	Typical	R168H + F295S		Prospective, KKI
		1.40	1,312.7				Prospective, KKI
CVN-101	1364	0.17	1,449	Typical	A305E		Prospective, KKI
CVN-102	102-101-004	0.63	1,908.7	Typical	A305E		Prospective, KKI
		0.90	2,509.2	Typical			Prospective, KKI
CVN-102	102-101-005	1.34	1,438.7	Typical	G27R		Prospective, KKI
		1.49	1,367.6				Prospective, KKI
CVN-102	102-101-008	2.04	1,344.4	Typical	A305E + G27R		Prospective, KKI
		2.14	1,623.3				Prospective, KKI

This table shows the source (literature, Natural History, or predose treatment trial) for individual urine NAA results along with their age and genotype, if available. The phenotype associated with each individual is also listed, along with whether the skill of walking/walking with aid was ever achieved. For the urine NAA results, if the method or laboratory was known it was listed, if not it was recorded as “unknown.” Urine NAA data from the natural history study and predose treatment trial individuals were recorded as retrospective or prospective. If the retrospective method and laboratory were known it was recorded, if not “unknown" was listed. All prospective urine NAA data were run at the central laboratory (KKI).

^a^ Genotypes referenced in Traeger, 1998 stating that Shaag, 1995 found these patients to be homozygous for A305E, however, the sex of Toft cases studies do not match up with the Shaag paper so the authors cannot be certain these participants are homozygous for A305E.

Cr, creatinine; GC, gas chromatography; KKI, Kennedy Krieger Institute; NAA, *N*-acetylaspartate.

Prospective natural history urine NAA samples were analyzed at the Kennedy Krieger Institute Biochemical Genetics Laboratory (henceforth referred to as the “central laboratory”) using a CLIA-validated stable isotope dilution gas chromatography/mass spectrometry method that has been previously described.^31^ The reference range is 0.7–26.7 mmol NAA/mol creatinine (Cr).

Retrospective urine NAA results from natural history study participants were taken from the medical records. The units used to report the Cr-normalized urine NAA varied; for comparative purposes, all results were converted to mmol/mol Cr where possible.

### Urine NAA from literature search

A literature search was conducted in PubMed using the search terms “mild CD,” “atypical CD,” “juvenile CD,” and “CD.” Literature was reviewed for quantitative urine NAA results, *ASPA* genotype, and description of phenotype. Not every publication listed quantitative urine NAA results. For those that did, the units used to report the Cr-normalized urine NAA varied; for comparative purposes, all results were converted to mmol/mol Cr units where possible.

In five publications, an exact NAA value was not provided and instead a “fold” change above normal levels was cited.^11,13,17,18,25^ In these cases, the upper end of the central laboratory reference range (26.7 mmol/mol Cr) was used for a conservative multiplier with the highest fold change value given. The case study from Kotambail et al. did not list the exact age for the patient when the urine NAA sample was collected. The age range given was 18–27 months.^7^ Conservatively, the age of 27 months was used in the analysis of age versus urine NAA levels.

### Genotype

Genotype information for the natural history study was available for 47/59 participants. The genotype information was obtained either from medical records or from genetic testing through Invitae or Fulgent Genetics using blood samples or buccal swabs to test for mutations in the *ASPA* gene.

### NAA statistical analyses

Microsoft Excel was used for calculations and to tabulate data for source, age, urine NAA result, genotype, and phenotype. GraphPad Prism version 10.1.2 was used for all graphs. A Mann–Whitney analysis was used for *t-*test comparisons between urine NAA results and phenotype groups. A pairwise *t-*test with a Benjamin–Hochberg multiplicity adjustment was used for age cohorts. The percent change for the longitudinal analysis utilized the first recorded urine NAA results as “baseline” and all subsequent results were compared with the baseline for that individual.

## RESULTS

### Differentiating between mild and typical CD phenotypes

Using combined prospective and retrospective CDC milestone data from the natural history study, accounting for age-appropriate milestones, 28/50 (56%) participants assessed for head control never achieved this skill, 30/43 (70%) did not achieve “sits without support,” and 28/36 (78%) never crawled. For the CDC milestones “pulls to stand/walks holding on” and “walks alone,” 83% (30/36) and 90% (35/39) of participants, respectively, did not achieve these skills (Supplementary Table S2). Of the participants that achieved “sits without support” (13/43) only 7 (53%) achieved walking/walking with assistance. This cohort of higher functioning participants, representing approximately 12% (7/59) of the natural history population, were outliers who achieved walking or walking with assistance (could “pull to stand and walk holding on”). These findings align with previous studies indicating that most patients with CD do not achieve sitting independently, and less than 10–20% display a milder disease phenotype with achievement of walking alone or with a mobility aid.^3,4^ Because a majority (80–90%) of patients with CD never achieve walking independently or with assistance, any individual with CD who achieved these skills was considered a “mild” phenotype for the purposes of this analysis. Any individual who never achieved walking, with or without assistance, was classified as having a “typical” phenotype. This strict skill achievement-based cutoff facilitated the analysis of the relationship between urine NAA levels and the two phenotypes, along with a subsequent analysis of the genotypes associated with milder phenotypes.

This phenotypic classification resulted in seven natural history participants being designated as “mild”: 1108, 1234, 1235, 1338, 1346, 1351, and 1357 (Table 1). Participant 1346 had no corresponding urine NAA result and therefore was not included in the rest of the analysis.

### Literature search

Applying the phenotypic cut-off to the literature search yielded 15 case studies describing 18 patients with mild CD.^5,7–14,17–22^ These 18 patients had all achieved or could be reasonably assumed to have achieved the skill of walking alone or walking with assistance, which would allow for them to be classified as “mild” for the purposes of this analysis (Table 1).

Seven published case reports (representing eight patients) included in the analysis provided data on typically progressing CD. Of these eight patients, only one was noted to have achieved sitting^30,32^ and none achieved walking.^25–29^

### Urine NAA and clinical phenotype (mild vs. typical)

**Figure 1.**
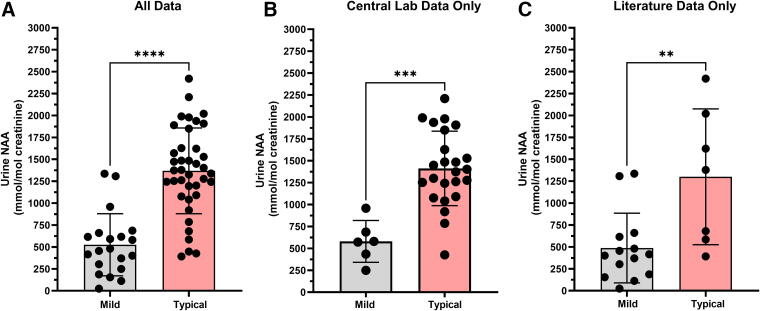
Urine NAA levels are different between mild and typical CD phenotypes. (**A**) Comparison of urine NAA levels utilizing all data (literature, retrospective, and prospective Natural History) demonstrates a significant difference (*p* < 0.0001) between mild (*n* = 20, mean 525.3 mmol/mol Cr, range 25.2–1,335 mmol/mol Cr) and typical phenotypes (*n* = 39, mean 1,369 mmol/mol Cr, range 391.7–2,420). (**B**) Restricting the comparison of urine NAA levels to results run only at the central laboratory still showed a significant difference (*p* = 0.0001) between phenotypes with mild (*n* = 6) having a mean of 580.4 mmol/mol Cr (range 249.5–958.4 mmol/mol Cr) and typical phenotypes (*n* = 24) having a mean of 1,412 mmol/mol Cr (range 426.2–2,209 mmol/mol Cr). (**C**) Restricting the comparison of urine NAA levels to only literature results showed a significant difference (*p* = 0.0074) between phenotypes with mild (*n* = 14) having a mean of 487.3 mmol/mol Cr (range 25.2–1,335 mmol/mol Cr) and typical phenotypes (*n* = 7) having a mean of 1,300 mmol/mol Cr (range 391.7–2420 mmol/mol Cr). CD, Canavan disease; Cr, creatinine; NAA, *N*-acetylaspartate.

Comparison of urine NAA levels between mild and typical phenotypes using results from the literature and the natural history study reveals a statistically significant difference (*p* < 0.0001) between the phenotypes (Fig. 1A). The urine NAA results for the mild phenotype group (*n* = 20) had a mean of 525.3 mmol/mol Cr (25.2–1,335 mmol/mol Cr). The typical phenotype group (*n* = 39) mean was 1,369 mmol/mol Cr (391.7–2,420 mmol/mol Cr). An acknowledged caveat to this analysis is the inclusion of different methodologies and laboratories from literature and retrospective natural history sources, which also may be a contributing factor to the overlap of urine NAA results between mild and typical phenotypes in Fig. 1A. Of note, all urine NAA results were stated to be pathological despite potential differences in methodology.

To address the result comparability issue, urine NAA results were reanalyzed by separating out the literature data from the natural history data reported from the central laboratory (Fig. 1B, C). The conclusions remained the same. For the natural history data only the mean of the mild CD phenotype group was lower than the typical phenotype group (*p* = 0.0001). Urine NAA results from the mild phenotype group (*n* = 6) had a mean of 580.4 mmol/mol Cr (249.5 to 958.4 mmol/mol Cr), while urine NAA results from the typical phenotype group (*n* = 24) had a mean of 1,412 mmol/mol Cr (426.2–2,209 mmol/mol Cr). Restricting the comparison of urine NAA levels to only literature results showed a significant difference (*p* = 0.0074) between phenotypes with mild (*n* = 14) having a mean of 487.3 mmol/mol Cr (range 25.2–1,335 mmol/mol Cr) and typical phenotypes (*n* = 7) having a mean of 1,300 mmol/mol Cr (range 391.7–2,420 mmol/mol Cr).

### Urine NAA and age effect

**Figure 2.**
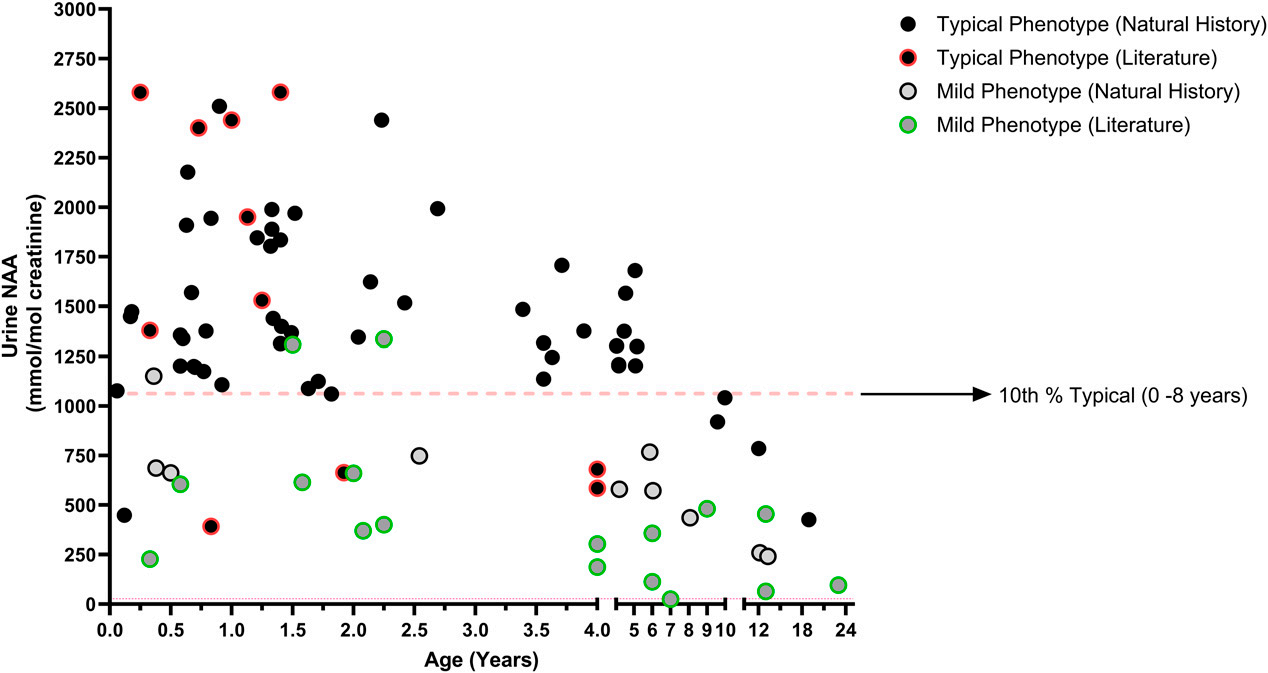
Scatter plot of urine NAA versus age. All urine NAA results were plotted vs age with phenotype denoted by filled black circles (typical) or filled gray circles (mild). Literature results are differentiated from Natural History results by having a colored outline to the circle, red for a literature result with a typical phenotype, and green for a literature result from a mild phenotype. Note that some individuals have multiple results represented on this plot, for longitudinal representation of each individual see Supplementary Figure S1. The red dashed line at 1,062 mmol/mol Cr represents the 10th percentile of all typical (Literature + Natural History) results from individuals between the ages of 0–8 years. Most urine NAA results from individuals classified with a mild phenotype fall below this 10th percentile line, while most individuals with a typical phenotype aged 0–8 years are above this line.

**Figure 3.**
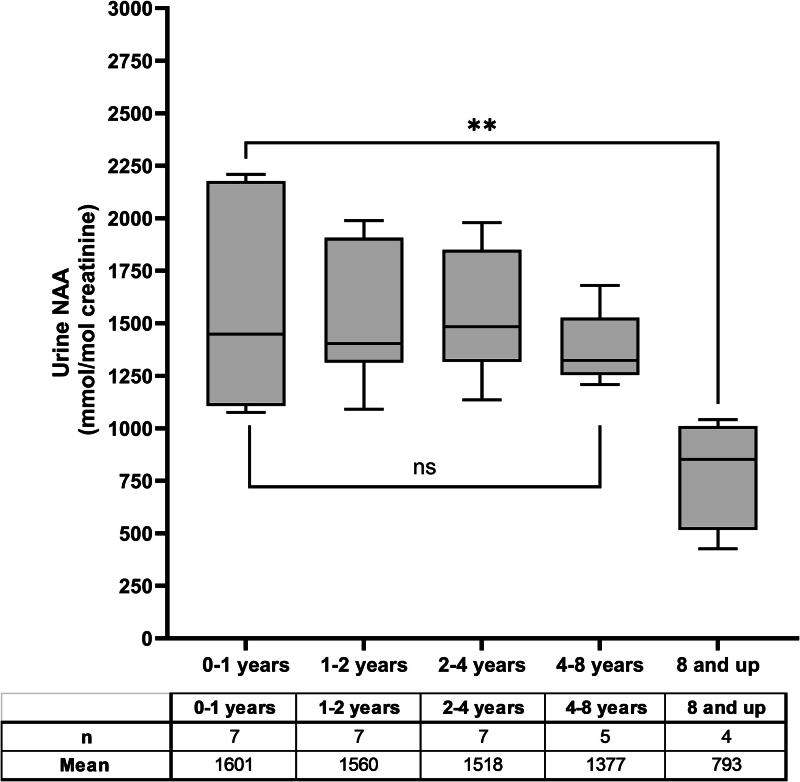
Age effect on urine NAA levels. Urine NAA results only from natural history participants with a typical phenotype whose sample was run at the central laboratory were used in this analysis. There is no statistical difference between the means of the selected age groups until age 8 years or older. The average from the 8 years and up group were lower, (*p* = 0.009) than the 0–1-year age category.

Urine NAA levels in both CD-affected and unaffected individuals have been reported to vary by age, with levels reported to decrease with age.^33,34^ A scatter plot of all urine NAA data demonstrates a clustering of mild phenotype results at ages above 8 years (Fig. 2, Supplementary Fig. S1). If urine NAA levels naturally decrease with age, this clustering of results in older individuals may have artificially inflated the statistical differences between the two phenotypes. Therefore, results were reanalyzed by age group using only typical phenotype participants from the natural history study with results from the central laboratory to ensure comparability (Fig. 3). The findings were similar to previous publications, with relatively stable NAA levels up to age 8 years.^33,34^ The age cohorts have means ranging from 793 to 1,601 mmol/mol Cr and *p*-values were not significant between any of the age groups up to 8 years old. After 8 years of age, a statistically significant decline in urine NAA levels was observed compared with the 0–1-year group (*p* = 0.0090), 1–2-year group (*p* = 0.0090), 2–4-year group (*p* = 0.0097), and 4–8-year group (*p* = 0.0498), albeit with limited data in the 8 years and older cohort (*n* = 4).

**Figure 4.**
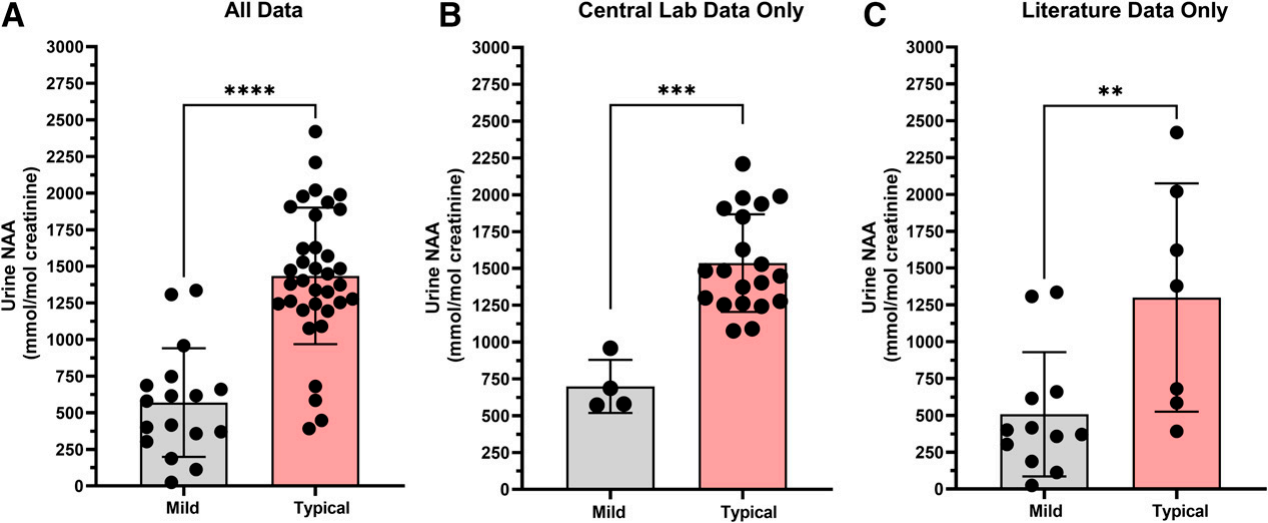
Age restricted to 0–8 years urine NAA comparison between mild and typical phenotypes. (**A**) Comparison of urine NAA levels utilizing all data and age restricted to 0–8 years demonstrates a significant difference (*p* < 0.0001) between mild (*n* = 17, mean 569 mmol/mol Cr, range 25.2–1,335 mmol/mol Cr) and typical phenotypes (*n* = 35, mean 1,434 mmol/mol Cr, range 391.7–2,420). (**B**) Restricting the comparison of urine NAA levels to results only run at the central laboratory still showed a significant difference (*p* = 0.0002) between phenotypes with mild (*n* = 4) having a mean of 699.4 mmol/mol Cr (range 572.1–958.4 mmol/mol Cr) and typical phenotypes (*n* = 20) having a mean of 1,536 mmol/mol Cr (range 1,076–2,209 mmol/mol Cr). (**C**) Restricting the comparison of urine NAA levels to only the literature results also show a significant difference (*p* = 0.0098) between phenotypes with mild (*n* = 12) having a mean of 507.4 mmol/mol Cr (range 25.2–1,335 mmol/mol Cr) and typical phenotypes (*n* = 7) having a mean of 1,300 mmol/mol Cr (range 391.7–2,420 mmol/mol Cr).

Due to the observed decline in urine NAA levels after 8 years of age, the mild versus typical phenotype comparison was reassessed. After restricting the comparison to ages 0–8 years, whether looking either at all data or just natural history data from the central laboratory, the conclusions remained consistent: urine NAA was lower, on average, in the mild phenotype group compared to the typical phenotype group. When using all data sources, the difference was statistically significant (*p* < 0.0001) with the mean mild phenotype urine NAA level (*n* = 17) at 569 mmol/mol Cr (range 25.2–1,335 mmol/mol Cr), and the mean typical phenotype level (*n* = 35) at 1,434 mmol/mol Cr (range 391.7–2,420 mmol/mol Cr) (Fig. 4A). When using just data from the natural history trial run at the central laboratory, the results were similar with a statistically significant difference (*p* = 0.0002) between the mean of the mild (*n* = 4) at 699.4 mmol/mol Cr (range 572.1–958.4 mmol/mol Cr), and the typical group (*n* = 20) at 1,536 mmol/mol Cr (range 1,076–2,209 mmol/mol Cr) (Fig. 4B). Restricting the comparison of urine NAA levels to only the literature results also shows a significant difference (*p* = 0.0098) between phenotypes with mild (*n* = 12) having a mean of 507.4 mmol/mol Cr (range 25.2–1,335 mmol/mol Cr) and typical phenotypes (*n* = 7) having a mean of 1,300 mmol/mol Cr (range 391.7–2,420 mmol/mol Cr) (Fig. 4C). The mean urine NAA in the mild phenotype group is >2-fold lower than the typical phenotype group. This reinforces the conclusion that, in aggregate, urine NAA can distinguish between phenotypes, and these differences are not due to age effect.

### Urine NAA intraindividual variability

**Figure 5.**
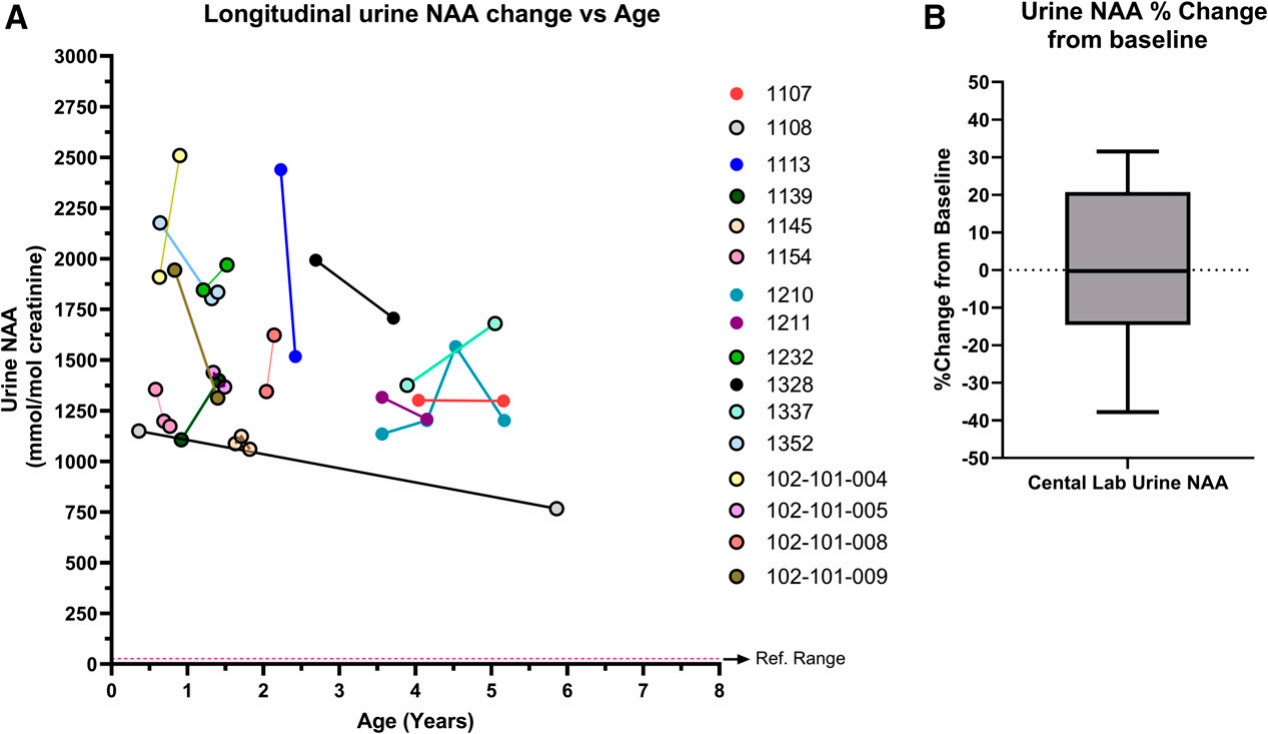
Intraindividual urine NAA variability over time. (**A**) Scatter plot of natural history participants, classified with a typical phenotype, with more than 1 measurement to show the variability of changes per individual. (**B**) Bar graph showing the percent change ranged from −37.8% to 31.5% with a mean change of −0.18%.

Understanding intraindividual changes in urine NAA levels is important for determining the natural variability of NAA levels and providing insight into meaningful decreases in the context of a clinical trial. Analyzing only natural history results from the central laboratory taken between 0 and 8 years old to avoid age and method-related confounders, 15 typical phenotype individuals and 1 mild phenotype individual had at least two results from samples taken on different days. Figure 5A demonstrates that although urine NAA levels can change over time, none of the changes in individuals with a typical phenotype result in levels <1,000 mmol/mol Cr. The only individual below 1,000 mmol/mol Cr on a subsequent measurement was 1108, who was classified as a mild phenotype. In Fig. 5B, the natural intraindividual variability ranged from −37.8% to 31.5% with a mean of −0.18% for those with a typical phenotype (Fig. 5A). For the one mild phenotype participant (1108), the change between measurements was −33.3%.

### Genotype/phenotype relationships

The differences in urine NAA levels between mild and typical phenotypes support the hypothesis that clinical outcomes may be dependent on residual ASPA activity. Investigation of the activity of the specific ASPA mutations was beyond the scope of this study; however, several of the mutations found in the mild phenotype group have been shown to have residual activity (Table 1).^15,35,36^ Two of those mutations are R71H and Y288C and were found in 56% (14/25) of individuals classified with a mild phenotype, in 0% (0/39) of the typical phenotype group.^10,13–15,17,^^19–22^ The G274R mutation was in 16% (4/25) of mild phenotype individuals, has a reported activity of 5–35%, and was not found in the typical phenotype group, however, it has been reported elsewhere as being associated with a typical phenotype.^8,11,15,35–37^ The A305E mutation was found in ∼40% of both mild and typical phenotype groups, reflecting previously published frequencies and the variable disease severity.^15,36,38^

## DISCUSSION

Due to the rarity and heterogeneity of CD it has been difficult to establish a relationship between urine NAA levels, genotype, and phenotype.^11,15,16,38^ The data analyzed herein are from a natural history study in CD and contributed a large number of comparable urine NAA results matched with genotype and phenotype; supplementing this with previously published case studies has allowed further characterization of the relationship between these variables. Using phenotype as a proxy for ASPA activity, the data demonstrate the ability of NAA to also reflect ASPA activity and delves into the natural variability of urine NAA levels in CD, laying the groundwork for the utilization of urine NAA beyond a diagnostic capacity.

When the average urine NAA levels of the mild or typical phenotype groups were compared, there was a statistically significant difference that remained regardless of age restriction and methodology. The 10th percentile of all typical phenotype results using all literature and natural history data from individuals aged 0–8 years was 1,062 mmol/mol Cr, or ∼1,000 mmol/mol Cr. The majority of the mild phenotype results were below this 10th percentile (Fig. 2). When looking at NAA results only from the natural history study run at the central laboratory to ensure method comparability, no results with a typical phenotype between ages 0 and 8 years old were below 1,000 mmol/mol Cr. These data demonstrate that urine NAA can distinguish between phenotypes, and if phenotype reflects residual ASPA activity, then NAA may also reflect ASPA activity.

To ensure that the differences in urine NAA levels between phenotypes were not due to natural variability, the effect of age and intraindividual variability was assessed. The results demonstrated no statistical differences in mean urine NAA levels between age groups until ages over 8 years old, where the mean NAA levels were statistically lower. This indicates that between 0 and 8 years, natural variability in urine NAA levels would not have accounted for the differences observed between the mild and typical phenotype groups.

Consideration needs to be taken when looking at NAA levels in children over 8 years of age, as NAA levels decrease after this time in both CD-affected and unaffected individuals.^33,34^ The decrease in NAA levels in children older than 8 years with CD could be attributed to brain atrophy seen in advanced disease.^24,39^ A recent study put forth that the atrophy could be driven by a mechanism in which neuronal cells are unable to differentiate and mature in the CD CNS environment.^40^ As neurons are the source of NAA in the brain, having fewer mature neurons as the disease progresses could account for the falling, though still pathological, NAA levels in these older children. This has important implications for the period in which urine NAA would be a useful biomarker for differentiating between phenotypes, tracking disease progress, or determining a drug effect during a clinical trial in older patients.

Intraindividual variability was also assessed to ensure that individuals with multiple measurements would have consistent results within the 0–8-year-old age bracket. Despite limitations of sample size, variable follow-up periods, and variable ages, none of the changes from baseline for the individuals with a typical phenotype resulted in NAA levels below the 1,000 mmol/mol Cr or exceeded a 40% decrease. These results have potential utility in a clinical trial setting using urine NAA as a biomarker, as levels <1,000 mmol/mol Cr are associated with better clinical outcomes (milder phenotypes). It would be unlikely to see a drop in urine NAA levels beyond 40% paired with a level <1,000 mmol/mol Cr without therapeutic intervention. However, it is unknown whether lowering NAA levels to the mild range will have a clinical effect, particularly in older children who have already accumulated significant CNS injury. Nonetheless, reducing urine NAA levels below 1,000 mmol/mol Cr would likely be indicative of a drug effect and may increase the probability of seeing clinical benefit.

While significantly lower average urine NAA levels were found between mild and typical phenotype groups, further investigation is needed regarding the use of NAA to distinguish phenotypes on an individual level for diagnostic purposes.

## CONCLUSION

Mean urine NAA levels in mild CD are greater than twofold lower than the typical phenotype. The natural variation of urine NAA levels within an individual and by age do not account for this difference between phenotypes. The lower NAA levels observed in mild CD were associated with specific genotypes that may have residual activity. Taken together these data support the hypothesis that urine NAA reflects ASPA activity and, at the population level, reflect phenotypic differences.

## ACCOUNTABILITY

Authors agree to be accountable for all aspects of the work, ensuring that questions related to accuracy or integrity are appropriately investigated and resolved.
