# Supplementary figures and images for: Urine N-Acetylaspartate Distinguishes Phenotypes in Canavan Disease
Source: Hum Gene Ther. 2025 Jan 16;36(1-2):45–56. doi: 10.1089/hum.2024.168 (PMC11807896; doi:10.1089/hum.2024.168)

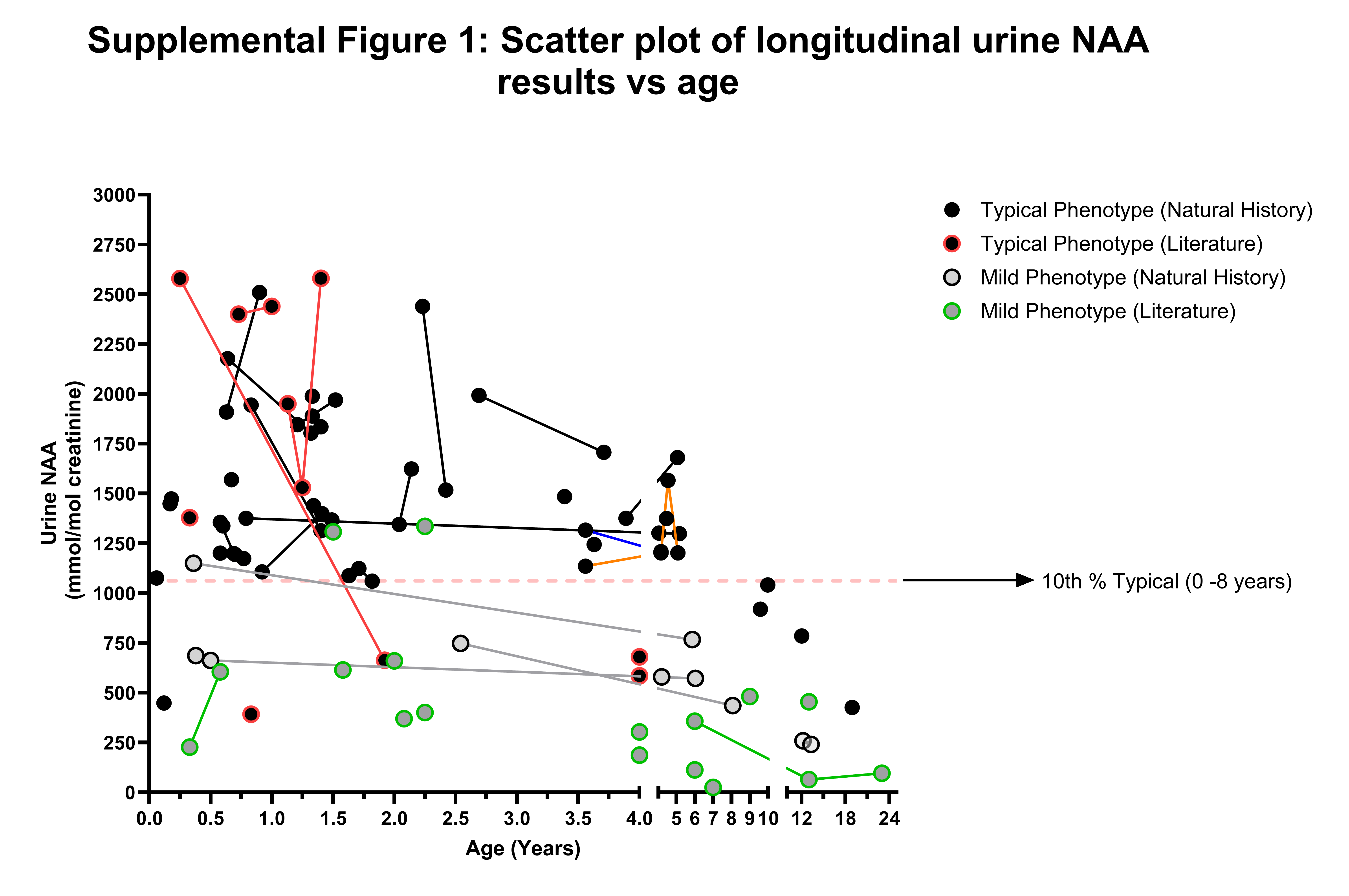

Supplement: Supplementary Figure S1 [file hum.2024.168_supp_figs1.tif]
