# Supplementary material for: Urine N-Acetylaspartate Distinguishes Phenotypes in Canavan Disease
Source: Hum Gene Ther. 2025 Jan 16;36(1-2):45–56. doi: 10.1089/hum.2024.168 (PMC11807896; doi:10.1089/hum.2024.168)
Supplement: Supplementary Table S1 [file hum.2024.168_supp_tables1.pdf]

Supplemental Table 1: Retrospective Natural History CDC Developmental Milestone Checklist

This checklist was used for the CDC milestone assessments for the retrospective analysis of natural history participant data.

Developmental Domain	CDC Developmental Milestone Checklist
**A. Social /Emotional and Cognitive**	
**Visual Attention**	4m Recognizes a familiar face
**Smiles**	2m Begins to smile at people
**Smiles**	4m Smiles spontaneously at people
**Play routines**	12m Plays games such as "peek-a-boo" and "pat-a-cake"
**Play routines**	9m Plays "peek-a-boo"
**Social Communication**	12m Uses simple gestures, like shaking head "no" or waving "bye-bye"
**B. Fine Motor/ Adaptive**	
**Tracking**	2m Begins to follow things with eyes
**Tracking**	4m Follows moving things from side to side
**Visual Attention**	Regards object
**Visual Attention**	9m Watches the path of something as it falls
**Reach**	4m Reaches for toy
**Hands to Mouth**	6m Brings things to mouth
**Hands to Mouth**	12m Hands you a book to read a story
**Grasp**	4m Can hold a small toy
**Grasp**	Raking grasp on small object
**Grasp**	9m Picks up things like cereal O's between thumb and index finger
**Cubes/Blocks**	6m Shakes and bangs a rattle
**Cubes/Blocks**	12m Puts things in a container, takes things out of a container
**Cubes/Blocks**	6m Begins to pass objects between hands
**Drawing**	18m Scribbles
**C. Language**	
**Receptive**	2m Turns head towards sounds
**Receptive**	6m Responds to own name
**Receptive**	12m Is shy or nervous with strangers
**Receptive**	9m Uses finger to point at things
**Receptive**	12m Looks at the right picture or thing when it's named
**Expressive**	2m Coos, makes gurgling sounds
**Expressive**	4m Babbles
**Expressive**	6m Strings vowels together ooh, eh and ah
**Expressive**	9m Copies sounds and gestures of others
**Expressive**	12m Uses simple gestures, like shaking head "no" or waving "bye-bye"
**Expressive**	6m Makes sounds to show joy and displeasure
**Expressive**	6m Begins to say consonant sounds (m and b)
**Expressive**	12m Says "mama" and "dada" and exclamations like "uh-oh!"
**Expressive**	12m Tries to say words you say
**D. Gross Motor**	
**Head Control**	4m Holds head steady unsupported
**Supine**	Moves arms and kicks
**Supine**	4m Brings hands to mouth
**Supine**	Rolls from back to sides
**Supine**	6m Rolls supine to prone

Developmental Domain	CDC Developmental Milestone Checklist
**Prone**	6m Prone to supine
**Prone**	2m Lifts head when on stomach
**Prone**	4m Pushes up to elbows
**Prone**	Pushes up on extended arms while on stomach
**Quadruped**	6m Rocks back and forth, may crawl backwards before forwards
**Quadruped**	9m Crawls
**Sit**	Pulls to sit no head lag
**Sit**	Sits with support at hips
**Sit**	6m Begins to sit momentarily without support
**Sit**	9m Sits without support
**Sit**	9m Can get into sitting
**Stand**	4m Pushes down on legs on a hard surface
**Stand**	6m When standing, supports weight on legs and may bounce
**Stand**	9m Stands holding on
**Stand**	12m Stands alone
**Stand**	12m Pulls up to stand, walks holding on to furniture ("cruising")
**Stand**	9m Pulls to stand
**Walk**	12m Takes a few steps alone
**Walk**	18m Walks alone
**Run**	18m Runs
**Stairs**	18m Walks up steps
