# Supplementary material for: Urine N-Acetylaspartate Distinguishes Phenotypes in Canavan Disease
Source: Hum Gene Ther. 2025 Jan 16;36(1-2):45–56. doi: 10.1089/hum.2024.168 (PMC11807896; doi:10.1089/hum.2024.168)
Supplement: Supplementary Table S2 [file hum.2024.168_supp_tables2.pdf]

Supplemental Table 2: Natural History Prospective and Retrospective CDC Developmental Milestone Data

CDC Milestone	Achieved	Never Achieved	Total
**Head Control**	22 (44%)	28 (56%)	50
**Sits Without Support**	13 (30%)	30 (70%)	43
**Crawls**	8 (22%)	28 (78%)	36
**Pulls to Stand/Walks Holding On**	6 (17%)	30 (83%)	36
**Walks Alone**	4 (10%)	35 (90%)	39

Achievement of the CDC milestones "head control", "sits without support", "crawls", "pulls to stand/walks holding on", and "walks alone" was marked as absent or present in either a retrospective data review or with an age-appropriate prospective assessment. Less than 20% of the natural history population were able to achieve "pulls to stand/walks holding on". Participants that were able to achieve this skill were classified as having a mild phenotype.
